# Understanding formation processes of calcareous nephrolithiasis in renal interstitium and tubule lumen

**DOI:** 10.1111/jcmm.18235

**Published:** 2024-03-20

**Authors:** Caitao Dong, Jiawei Zhou, Xiaozhe Su, Ziqi He, Qianlin Song, Chao Song, Hu Ke, Chuan Wang, Wenbiao Liao, Sixing Yang

**Affiliations:** ^1^ Department of Urology Renmin Hospital of Wuhan University Wuhan Hubei Province China

**Keywords:** abnormal urine, calcium‐containing stones, inflammation, nephrolithiasis, oxidative stress, pathogenesis, Randall's plaque, Randall's plug

## Abstract

Kidney stone, one of the oldest known diseases, has plagued humans for centuries, consistently imposing a heavy burden on patients and healthcare systems worldwide due to their high incidence and recurrence rates. Advancements in endoscopy, imaging, genetics, molecular biology and bioinformatics have led to a deeper and more comprehensive understanding of the mechanism behind nephrolithiasis. Kidney stone formation is a complex, multi‐step and long‐term process involving the transformation of stone‐forming salts from free ions into asymptomatic or symptomatic stones influenced by physical, chemical and biological factors. Among the various types of kidney stones observed in clinical practice, calcareous nephrolithiasis is currently the most common and exhibits the most intricate formation mechanism. Extensive research suggests that calcareous nephrolithiasis primarily originates from interstitial subepithelial calcified plaques and/or calcified blockages in the openings of collecting ducts. These calcified plaques and blockages eventually come into contact with urine in the renal pelvis, serving as a nidus for crystal formation and subsequent stone growth. Both pathways of stone formation share similar mechanisms, such as the drive of abnormal urine composition, involvement of oxidative stress and inflammation, and an imbalance of stone inhibitors and promoters. However, they also possess unique characteristics. Hence, this review aims to provide detailed description and present recent discoveries regarding the formation processes of calcareous nephrolithiasis from two distinct birthplaces: renal interstitium and tubule lumen.

## 1 INTRODUCTION

The earliest known human record of kidney stones dates back to an Egyptian mummy from around 4800 BC.^1^ Throughout history, many cases of kidney stones were also documented by the ancient Greeks and Romans, with Hippocrates himself describing the symptoms and treatment of the condition.^2^ Patients suffering from symptomatic kidney stones experience intense pain in the back, side, lower abdomen, groin and/or gross haematuria, often accompanied by nausea, vomiting or sweating.^3^, ^4^ In severe cases, nephrolithiasis can lead to complications such as hydronephrosis, acute and/or chronic kidney disease, sepsis and other serious medical conditions,^5^, ^6^ which can significantly reduce an individual's quality of life and even be life‐threatening.^7^ Still, nephrolithiasis remains a global issue affecting individuals of all ages, races, cultures and regions. In recent years, there has been a dramatic increase in its incidence, particularly in developed countries. The rising prevalence of kidney stones substantially burdens healthcare systems worldwide.^8^, ^9^, ^10^ In the United States alone, the total expenditure for kidney stone treatment was approximately $2.1 billion in 2000, which has since risen to an estimated $3.79 billion. This expenditure is projected to reach another $1.24 billion annually by 2030,^11^ further exacerbating the burden on the global healthcare system.^12^

Currently, advancements in medical technology have provided significant benefits to patients, and surgical removal is the most commonly used and effective therapies treatment for kidney stones. Procedures such as extracorporeal shock wave lithotripsy (ESWL), ureteroscopy and percutaneous nephrolithotomy (PCNL) have proven successful.^13^, ^14^ However, the high recurrence rate of kidney stones, with approximately half of the patients forming new stones radiographically or experiencing symptoms, has long puzzled urologists and researchers.^15^, ^16^, ^17^ Surgical treatments primarily address symptoms rather than offering a definitive cure for the disease. Medication therapies, such as thiazides,^18^ alkaline citrate,^19^ allopurinol and other medications, are widely recommended by guidelines and employed in clinical practice.^20^, ^21^ However, there have been limited updates in recent decades regarding the development of new and more effective medications. Furthermore, fluid intake and dietary modifications, such as consuming an ample amount of water and making dietary adjustments to reduce the consumption of certain foods that contribute to kidney stone formation, represent a cost‐effective strategy for both individuals and society.^22^, ^23^ However, managing kidney stones through diet alone by targeting a single risk factor may not yield optimal results in achieving the desired protective effect. Therefore, gaining a more comprehensive understanding of the mechanisms and risk factors involved in kidney stone formation could contribute to developing novel therapies for stone prevention.

Through years of dedicated research, significant progress has been made in understanding the formation processes of nephrolithiasis. The formation of kidney stones is a complex, multi‐step and long‐term process influenced by physical, chemical and biological factors that transform stone‐forming salts from free ions into asymptomatic or symptomatic stones.^7^, ^24^, ^25^ From a macroscopic perspective, stone formation is influenced by various factors such as genetic predisposition,^26^, ^27^ environmental and geographic conditions,^28^, ^29^, ^30^ gender,^31^, ^32^ age^33^ and individual differences, including dietary habits^8^, ^34^ and income levels.^35^, ^36^ On a microscopic level, the process of stone formation is mediated by factors like urine supersaturation with stone‐forming salts,^37^ the duration of abnormal urine residence, urine pH values,^38^ and the imbalance of stone inhibitors and promoters.^38^, ^39^, ^40^ Kidney stones are primarily categorized based on their mineralogical composition, including calcium stones, uric acid stones, cystine stones and struvite stones.^7^ Among these, calcium stones, specifically composed of calcium oxalate (CaOx) and/or calcium phosphate (CaP, calcium hydroxyapatite or brushite), account for over 80% of all types of kidney stones.^41^, ^42^ Thus, this review focuses on comprehensively understanding the pathophysiological processes involved in calcium stone formation, prioritizing the exploration of treatments aimed at calcareous nephrolithiasis, thereby reflecting the urgency in developing effective strategies for kidney stone prevention.

**FIGURE 1:**
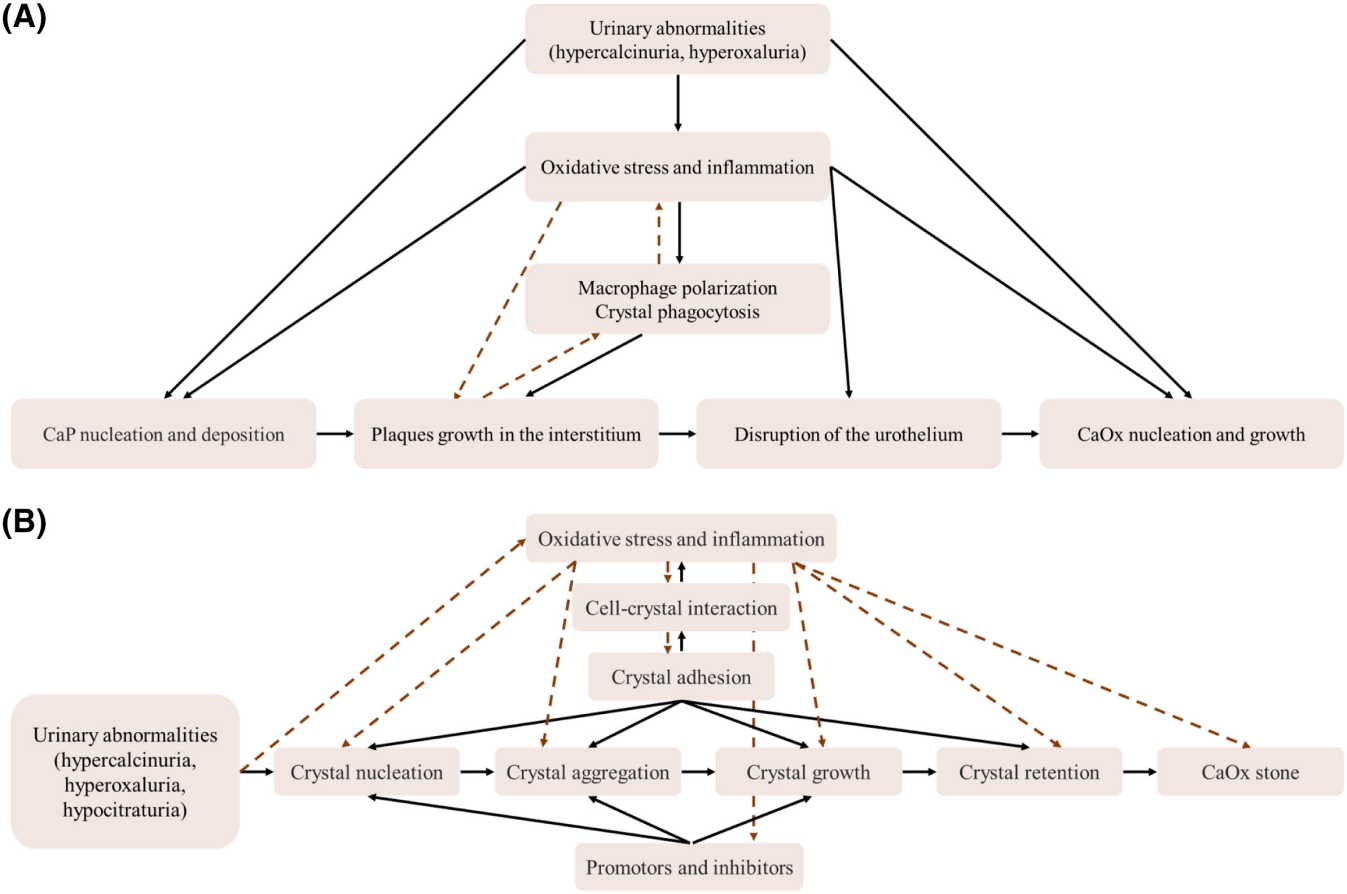
Flow chart showing the formation processes of calcareous nephrolithiasis in renal interstitium and tubule lumen. (A) The formation processes of calcareous nephrolithiasis in renal interstitium. In the first step, CaP is initially deposited in the basement membranes of the thin Henle loops under conditions of abnormal urine. The deposited CaP aggregates and grows within the papilla interstitium until the plaque reaches and breaks through the urothelium. Then, the exposed plaque acts as a nidus for the crystallization and growth of CaOx stones under conditions of calcium oxalate supersaturation in the urine. During the formation of plaques, CaP deposits can be regarded as foreign bodies to trigger the inflammatory response and the involvement of macrophages. In addition, oxidative stress and inflammatory response can occur in renal tubular epithelial cells, renal interstitial cells, renal immune cells and urothelial cells under the irritation of abnormal urine and plaques, thus involving all steps of RP formation. (B) The formation processes of calcareous nephrolithiasis in renal tubular lumen. Under the conditions of abnormal urine, stone formation in renal tubular lumen mainly undergoes crystal nucleation, growth, aggregation and adhesion, with the involvement of various promotors and inhibitors. Among them, crystal adhesion is closely related to each step of the formation of Randall's plugs. Additionally, cell‐crystal interaction can lead to oxidative stress and inflammation in RTECs, which in turn promote stone formation.

In 1937, Alexander Randall made a significant contribution to the understanding of kidney stone formation through histopathological examination of cadavers' kidneys. He proposed that the initial lesions for kidney stone formation could be found on the renal papilla and identified two types: interstitial subepithelial calcified plaques (currently known as ‘Randall's plaques’) and calcified deposits in the collecting ducts (currently known as ‘Randall's plugs’).^43^ These observations laid the foundation for current theories regarding the birthplace of calcium stones. In this review, we provide a comprehensive description of the origins and development of calcium stones, presenting from two perspectives: in renal interstitium and tubule lumen (Figure 1). We aim to enhance the understanding of calcareous nephrolithiasis among urologists and researchers in this field. Additionally, we present an overview of the current research status of calcareous nephrolithiasis, contributing to ongoing efforts to expand knowledge and improve treatment strategies for this condition.

## 2 ABERRANT SOLUTE CONCENTRATIONS IN URINE

Aberrant concentration of stone‐related salts plays a critical role in driving each step of stone formation.^37^ Hypercalciuria, hyperoxaluria and hypocitraturia have consistently been identified as important factors in the aetiology of urolithiasis, as shown in our previous bibliometric study.^45^ Table 1 summarizes some of the pathophysiological causes, dietary habits, common disorders and genetic factors involved in the mechanisms of calcium‐containing stone formation. It is well‐known that reduced urine volume can lead to an increased concentration of solutes, making stone formation more likely. Therefore, it is generally recommended to maintain a high fluid intake to prevent stone formation.^46^, ^47^ Urinary calcium concentration is a major determinant of the supersaturation of CaP and CaOx. Hypercalciuria, characterized by elevated urinary calcium levels, is the most common abnormality in stone‐forming patients, affecting 30%–60% of individuals.^48^ Hypercalciuria can have various causes, including excessive animal protein intake, dietary acid or sodium,^47^, ^49^ hyperparathyroidism,^50^ chronic metabolic acidosis^51^, ^52^ and certain monogenic disorders (such as Dent disease, Bartter syndromes, abnormalities of the calcium‐sensing receptor and so on).^53^ Increased gut absorption of calcium, increased bone resorption and decreased renal tubular reabsorption can all contribute to elevated urinary calcium levels. The association between dietary calcium intake and the risk of stone formation has been a topic of controversy in the past, mainly due to concerns about hypercalciuria. However, recent clinical studies have provided evidence supporting the beneficial effect of dietary calcium supplementation in reducing the risk of calcium stone formation.^54^, ^55^, ^56^, ^57^ The European Association of Urology (EAU) guidelines recommend a daily dietary calcium intake of 1000–1200 mg for individuals with recurrent CaOx stone formation.^20^, ^48^

**TABLE 1:** The causes of hypercalciuria, hyperoxaluria and hypocitraturia in patients with calcareous nephrolithiasis.

Abnormal urine	Hypercalciuria^52^, ^72^, ^73^	Hyperoxaluria^24^, ^48^, ^58^	Hypocitraturia^48^, ^53^, ^70^
Pathophysiology	Increased intestinal calcium absorption; reduced renal tubular reabsorption; increased bone resorption	Increased oxalate absorption from food; increased production of endogenous oxalate; diminished binding of oxalate by intestinal luminal calcium	Dysregulation of the acid–base balance in kidney; reduced excretion of citrate in acid loads
Dietary habit	Low fluid intake; high sodium intake; high glucose or sucrose intake; excess of animal protein or acid intake; overuse of calcium supplements and vitamin C	Low fluid intake; excess of oxalate‐rich foods intake (such as Vitamin C, chocolate, nuts, rhubarb, spinach, beetroot, almond and starfruit); severe dietary calcium restriction; a high‐protein diet	Excess of animal protein or acid in diet; high sodium intake
Common disorders	Primary hyperparathyroidism; Vitamin D intoxication; immobilization; chronic metabolic acidosis; secondary distal tubular acidosis (dRTA)	Roux‐en‐Y gastric bypass; exocrine pancreatic insufficiency; fat malabsorption (such as short bowel syndrome, coeliac disease, Crohn's disease and cystic fibrosis); the use of medications (such as octreotide and orlistat)	Alkali loss (such as intestinal malabsorption or chronic diarrhoea); metabolic acidosis from any cause; the use of carboanhydrase inhibitors (such as topiramate and acetazolamide); potassium depletion (such as Thiazide diuretics); secondary distal tubular acidosis (dRTA)
Genetic variants	Dent disease (CLCN5 mutation); Bartter syndromes (NKCC2 and ROMK mutation)	Primary hyperoxaluria type I (AGXT mutation)	Hereditary dRTA (SLC4A1, ATP6V1B1 or ATP6V0A4, FOXI1, WDR72 and CA2 mutation); bbTT polymorphism of the vitamin D receptor
	Abnormalities of the calcium‐sensing receptor (CaSR); Familial hypomagnesemia hypercalciuria syndrome (CLDN16 mutation); other claudin family of genes (particularly CLDN10, CLDN14, CLDN19) mutation; Familial hyperkalemic hypertension (WNK1 or WNK 4 mutation); NHE3 mutation	Primary hyperoxaluria type II (GRHPR mutation); primary hyperoxaluria type III (HOGA1 mutation); SLC26A6 deficiency	
	TRPV5 mutation; Klotho deficiency; hereditary dRTA (SLC4A1, ATP6V1B1 or ATP6V0A4, FOXI1, WDR72 and CA2 mutation); Pseudoxanthoma elasticum (PXE, ABCC6 mutation)		

Abbreviations: ABCC6, ATP binding cassette subfamily C member 6; AGXT, alanine–glyoxylate aminotransferase; ATP6V1B1 and ATP6V0A4, B1 and a4 subunits of the V‐ATPase; CA2, carbonic anhydrase type II; CLDN, claudin family of genes; FOXI1, Forkhead Box I1; GRHPR, glyoxylate reductase/hydroxypyruvate reductase; HOGA1, 4‐hydroxy‐2‐oxoglutarate aldolase type 1; NHE3, sodium/proton exchanger 3; NKCC2, Na‐K‐Cl cotransporter type 2; ROMK, inward‐rectifier potassium channel; SLC26A6, the solute carrier family 26 member 6; SLC4A1, solute carrier family 4 member 1; TRPV5, transient receptor potential vanilloid 5; WDR72, tryptophan aspartate repeat domain 72; WNK, serine/threonine‐protein kinase.

Hyperoxaluria is a significant contributing factor to the supersaturation of CaOx in urine.^53^ Urinary oxalate concentration is primarily influenced by the absorption of oxalate in the bowel or its endogenous metabolism in the liver.^48^ In the gastrointestinal tract, conditions that reduce the binding of oxalate and calcium, such as excessive intake of oxalate‐rich foods intake (e.g., spinach, cocoa, beets, peppers, chocolate and nuts), severe dietary calcium restriction or intestinal malabsorption (due to small bowel disease, intestinal resection or bypass), can increase oxalate absorption.^24^, ^37^ Dysfunction in key steps of the oxalate biosynthetic pathway can also result in significantly higher oxalate concentrations in urine, as seen in primary hyperoxaluria (I, II and III).^58^ Although there is limited evidence demonstrating the efficacy of low dietary oxalate intake in reducing the risk of CaOx stone formation, it is still recommended for individuals with CaOx stones to control their intake of oxalate‐rich foods.^48^ The presence of *Oxalobacter formigenes*, a member of the normal gut microflora that utilizes oxalate as a food source, can influence the enteric absorption of oxalate.^59^ Several studies have shown that colonization with *Oxalobacter formigenes* reduces the risk of CaOx stone recurrence^59^ and treatment with *Oxalobacter formigenes* prevents CaOx deposition in the kidneys of rats.^60^ However, a few studies have reported that oral administration of *Oxalobacter formigenes* did not obviously influence urinary oxalate excretion levels.^61^, ^62^ Ticinesi and colleagues using 16S rRNA microbial profiling discovered a larger population of oxalate‐degrading microflora that exhibited a negative correlation with urinary oxalate concentration and further proposed a hypothesis suggesting that the presence of a gut–kidney axis influences the development of CaOx stone formation.^63^ Stepanova and colleagues further explored this hypothesis and reported that disrupting the native gut flora through Ceftriaxone treatment significantly increased urinary oxalate levels. Conversely, rats treated with synbiotics, which promote the growth of beneficial gut microbes, showed a significant decrease urinary oxalate excretion. Based on these findings, manipulating the gut–kidney axis to alleviate hyperoxaluria has emerged as a potentially promising therapeutic approach for patients with CaOx stones.^64^

In addition to hypercalciuria and hyperoxaluria, hypocitraturia is strongly associated with stone formation and is present in approximately 20%–60% of patients with calcium stones.^65^, ^66^, ^67^ Urinary citrate plays a crucial role in inhibiting the formation of calcium stones by binding with urinary calcium, thereby reducing the concentration of ionized calcium and interfering with calcium crystal nucleation and growth.^68^, ^69^ The body's acid–base status significantly influences urinary citrate excretion, with the pH of proximal tubule cells being a key regulator. Acidosis increases the reabsorption and metabolism of citrate in renal tubular epithelial cells, while alkalosis decreases these processes.^24^, ^48^, ^70^ Potassium citrate is commonly used in clinical practice to prevent stone recurrence, although the exact pathological factors underlying decreased urinary citrate levels in most calcium stone formers with hypocitraturia are still not well understood.^65^ Fruits and vegetables rich in citrate or alkali are often advised for stone formers as they may increase urinary citrate levels. However, a PROBE trial reported that daily supplementation of lemon juice did not significantly increase citrate excretion or reduce the risk of stone recurrence.^71^ Furthermore, other urinary abnormalities, such as hyperuricosuria, not only contribute to the formation of uric acid stones but also reduce the solubility of calcium oxalate, promoting the formation of calcium oxalate stones.^24^, ^53^

## 3 OXIDATIVE STRESS AND INFLAMMATION

Over the years, despite treatments aimed at reducing urinary supersaturation of stone‐related salts, approximately 50% of patients will still experience stone recurrence within the next 5 years.^33^, ^74^ This recognition has led to a growing understanding that stones result from complex interactions between various cells, including renal epithelial cells, renal interstitial cells and other, in response to the abnormal urinary environment and the presence of RPs. Oxidative stress and inflammation have emerged as important factors in the pathogenesis of urolithiasis.^75^ The association between oxidative stress, characterized by an imbalance between reactive oxygen species and antioxidant defences, and the formation of kidney stones has been a topic of intense research in recent years. Oxidative stress and inflammation are mainly involved in multiple steps along the possible mechanisms of stone formation, including injury and death of tubular epithelial cells,^76^ epithelial‐to‐osteoblast transformation,^7^ calcification of the vasa recta,^75^ collagen mineralization,^77^ macrophage recruitment and crystal clearance,^78^ the disruption of the urothelium^79^ and production of various crystallization modulating macromolecules.^38^ Clinical data have demonstrated that patients with calcium oxalate stones have higher level of injury markers (such as variety of renal enzymes, thiobarbituric acid‐reactive substances and urinary 8‐hydroxydeoxyguanosine), inflammatory and proinflammatory cytokines (such as MCP‐1, CCL5, CCL7, IL1β, TNF, IL6, IL8 and M‐CSF) and lower antioxidant levels (such as α‐carotene, α‐carotene and β‐cryptoxanthin) in their urine.^75^, ^80^ Renal papilla specimens from patients with RPs exhibit extensive cell damage and interstitial fibrosis surrounding the crystal blockages, with the stone matrix containing inflammation‐related proteins.^40^, ^81^ Basic studies examining renal epithelial cell responses to high concentration of oxalate or CaOx crystals and animal models of hyperoxaluria‐induced CaOx crystal deposition have further highlighted the importance of oxidative stress and inflammation.^82^, ^83^, ^84^ Overwhelming oxidative stress and inflammatory responses can lead to the expression of molecules involved in crystal binding, disruption of tight junctions, injury, cell death and shedding in RTECs.^77^, ^82^ Additionally, oxidative stress and inflammation mediate the polarization of macrophages, collagen mineralization, macrophage‐mediated stone clearance, interstitial fibrosis and the formation of RPs in the kidney interstitium.^73^, ^85^ Notably, most macromolecular modulators of crystallization are inflammatory mediators produced via redox‐dependent signalling pathways.^73^, ^76^ In a recent study, Canela and colleagues employed an integrated multi‐omics analysis to examine the renal papillae in individuals with kidney stones. Their findings revealed the involvement of immune inflammation and oxidative stress in mineral deposition, and suggested that MMP7 and MMP9 could serve as potential biomarkers for the detection of kidney stones.^86^ Considering these pieces of evidence, it is clear that oxidative stress, inflammatory responses and crystallization interact with each other, forming a vicious cycle that ultimately leads to the formation of ‘end products’. Consequently, reducing oxidative stress and inflammatory responses in the kidneys of stone formers holds potential as a new therapeutic approach.

## 4 THE FORMING PROCESSES OF CALCIFIED PLAQUES IN RENAL INTERSTITIUM

**FIGURE 2:**
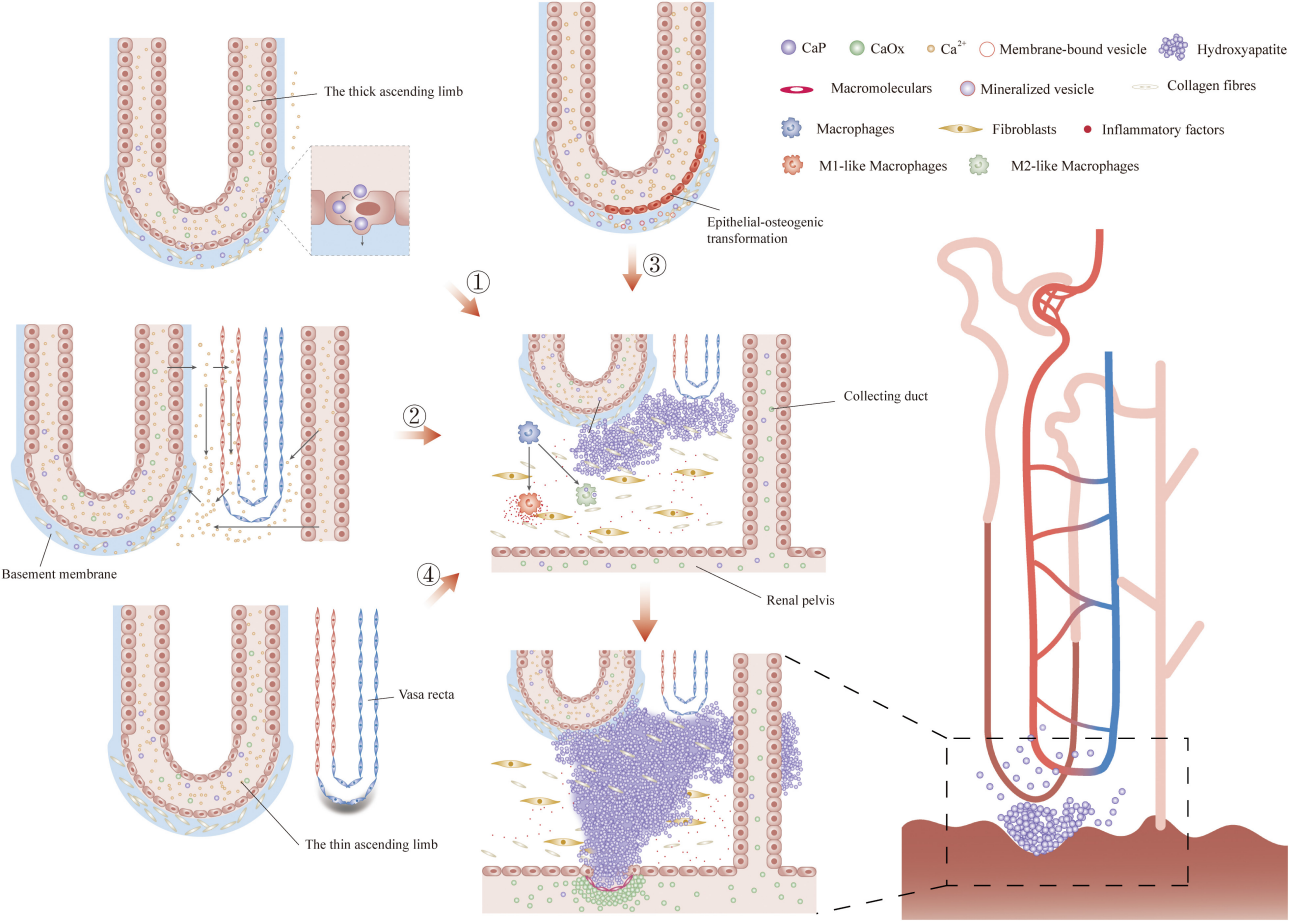
The forming processes of calcified plaques in renal interstitium. There are four main hypotheses regarding how the crystal deposits first appeared in the renal interstitium: ① CaP crystals formation occurs in the loop of Henle, and CaP crystals enter directly into the exposed basal membrane due to cell damage or defective tight junction or are endocytosed by cells and then exocytosed to the basal membrane side. ② Excessive calcium ions in the Henle loops are reabsorbed in the thick ascending limbs and enter the bloodstream, leading to an increase in calcium ion concentration in the renal inner medulla. ③ Prolonged exposure of epithelial cells to abnormal urine induces them to acquire an osteogenic phenotype, resulting in the production of matrix vesicles that provide suitable sites for CaP deposition. ④ Injury and repair of the vasculature at the renal papillary tip lead to atherosclerotic‐like calcification of the vasa recta. Once CaP crystals deposit in the basement membrane of the Henle loops, the deposition continues to grow. CaP deposits act as a foreign body and trigger an immune response in the kidney. Macrophages may play a role in clearing small crystals. As the plaque persistently grows in the renal interstitium, it eventually reaches and breaks through the urothelium. Subsequently, the exposed calcified plaque comes into contact with the urine and becomes covered by macromolecules. Ultimately, the naked plaque serves as a nidus for the crystallization and growth of calcium oxalate stones under conditions of calcium oxalate supersaturation.

The interstitial subepithelial calcified plaques (also termed Randall's plaque, RP) have been observed in a significant proportion of idiopathic CaOx stone formers, contributing around 80% of all stone formers without underlying systemic disorders.^75^, ^87^ Anderson and McDonald firstly identified interstitial crystal deposits using microscope in the papilla of autopsied kidneys.^88^ Stoller and colleagues conducted a study using high‐resolution radiography and found that subepithelial RPs were present in 57% of cadaveric kidneys.^89^ In a cross‐sectional study involving 30,149 intact stones, predominantly composed of CaOx, visible traces originating within RPs were detected in 34.1% of the stones.^32^ Additionally, Michael P. and colleagues reported that under endoscopic observation during percutaneous nephrolithotripsy, 98.7% of patients (77 of 78) had at least one RP in the renal papilla.^90^ Interestingly, RPs have been identified not only in stone formers but also in 16 out of 22 non‐stone formers, suggesting the presence of incipient RPs.^91^ In our previous bibliometric study, it was found that research on the origin of RPs invariably has consistently been a prominent area of focus in this field over the past three decades.^45^ Understanding these formation processes of interstitial calcified plaques is not small task. The prevailing theory of calcium stone formation involves a two‐step process, as depicted Figures 1A and 2. In the first step, CaP is initially deposited in the basement membranes of the thin Henle loops, forming a mass of CaP aggregates within the renal papilla interstitium, eventually developing into a larger CaP plaque that breaks through the urothelium. The second step involves the exposed CaP plaque acting acts as a nidus for crystallizing and growing CaOx stones under conditions of calcium oxalate supersaturation in the urine.^92^

### 4.1 The possible theories about the initial stages of calcified plaque formation in renal interstitum

#### 4.1.1 CaP deposition in basement membranes

In 2002, Andrew P. Evan published a significant finding indicating that the basement membranes of the thin Henle loops were indeed the sites of origin for RPs.^81^ In 2018, Andrew P. Evan further proposed that RPs specifically originate in the thin ascending Henle loops.^93^ The process of CaP precipitation in basement membranes involved the following mechanism: The basement membranes of the thin Henle loops are relatively thick and primarily composed of collagens, which along with a substantial presence of mucopolysaccharides, and provide numerous electrostatically binding sites for calcium and phosphate ions.^81^

As there was evidence that CaP supersaturation, occurring in the thin Henle loops, adequately induced CaP crystallization,^94^ and a few studies have reported the endocytosis of crystals by tubular epithelial cells,^95^, ^96^, ^97^ thus there exist arguments that CaP crystals, originating in the tubular lumen, may be endocytosed and exocytosed by cells and then deposit in basement membranes.^77^, ^91^, ^94^ Additionally, impaired functions of the tight junction, impairing paracellular permeability and barrier function, may be involved in the translocation of crystals.^98^ Paleerath and Thongboonkerd^98^ demonstrated that calcium oxalate monohydrate (COM) crystals could reduce the expression of zonula occludens‐1 (ZO‐1), leading to the disruption of tight junctions in renal tubular epithelial cells, which may occur due to the relocalization of alpha‐tubulin^99^ or reorganization of F‐actin in these cells.^100^ Furthermore, abnormal urine composition can induce death and sloughing of renal tubular epithelial cells, exposing the basement membrane directly to urine.^101^ However, observations using light and electron microscopy of renal papillae from individuals with RPs have shown no significant presence of crystals in the tubular lumen or cells of the thin Henle loops. Additionally, the cells showed no signs of damage. There findings strongly indicate that CaP may precipitate directly in basement membranes rather than traversing across epithelial cells.^81^, ^91^

Calcium phosphate (CaP) deposition is primarily driven by relevant solutes in the lumen or interstitial fluid.^25^, ^48^ Hypercalciuria is a well‐known risk factor for calcium stones, and interstitial calcium accumulation also plays a crucial role in CaP deposition.^75^, ^102^, ^103^ Previous studies, such as the comprehensive review by Alexander, have extensively discussed the perspectives on calcium transport from renal tubules.^52^ After glomerular filtration, approximately 60%–70% of urinary calcium ions are reabsorbed in the proximal convoluted tubules. Subsequently, 20%–25% and 10%–15% of the remaining urinary calcium ions are reabsorbed in the Henle loops and distal convoluted tubules.^104^, ^105^ In the case of calcium stone formers with idiopathic hypercalciuria, Worcester and colleagues found that increased sodium and calcium ions were delivered to the Henle loops compared to normal subjects.^106^ Intriguingly, the presence of considerable AQP1 in the thin descending Henle loops, which primarily facilitate water reabsorption but have limited solute permeability, further enhances the supersaturation of tubular calcium and phosphate ions.^102^ Consequently, the increased calcium concentration may elevate the calcium reabsorption level in the thick ascending limbs or the inner medullary collecting ducts, leading to the accumulation of interstitial calcium. The concept of ‘vas washdown’ explains this phenomenon better. In the outer medulla, a certain number of thick ascending limbs surround the descending vas recta. Increased calcium reabsorption, but without concurrent water permeability, in the thick ascending limbs promotes the passive diffusion of more calcium ions into the descending vas recta, which subsequently returns to the deep medulla.^93^ While calcium is a key factor, it is likely that interstitial pH and phosphate levels also influence CaP deposition. However, research specifically addressing the impact of these factors has been limited.

Another hypothesis suggests that CaP deposits in basement membranes may develop from membrane‐bound matrix vesicles produced by epithelial cells, which acquire an osteogenic phenotype when exposed to abnormal urine.^7^, ^77^ This hypothesis draws upon the process of bone matrix mineralization, where the entry or accommodation of calcium and phosphate into matrix vesicles, derived from chondrocyte or osteoblast membranes, leads to the formation of amorphous calcium phosphate and subsequent production of dense hydroxyapatite through complex steps.^107^ Examination of renal papillae tissues from stone formers revealed that primary CaP deposits in basement membranes were associated with membrane‐bound matrix vesicles. Furthermore, spherical CaP deposits containing needle‐shaped apatitic crystals were observed, suggesting the presence of calcified vesicles.^73^, ^108^ Based on these observations, researchers propose that the abnormal urinary environment in stone formers, such as hypercalciuria, hyperoxaluria and hypocitraturia, induces an epithelial‐to‐osteoblast transition. These specific epithelial cells may produce matrix vesicles similar to those involved in bone mineralization, providing feasible sites for CaP deposition.^73^ Several in vitro and in vivo studies provide support for this hypothesis, demonstrating up‐regulation of osteogenic markers, including BMPs, RUNX2, Osterix, Osteonectin, Osteopontin and Alkaline Phosphatase (ALP), in oxalate‐induced renal tubular epithelial cells (RTECs) and rat models of hyperoxaluria.^109^, ^110^, ^111^, ^112^, ^113^ While the presence of vesicles and certain osteogenic‐related proteins in stone components has been observed, direct histological evidence of epithelial cells in Henle loops or other nephrons acquiring an osteoblast‐like phenotype in stone formers is limited.

#### 4.1.2 The origin of calcified plaques from vascular calcification

Marshall L. Stoller found that a small number of plaques also appeared in the basement membranes of the vasa recta.^89^ Furthermore, epidemiological studies have established an association between cardiovascular disease and kidney stones,^114^, ^115^, ^116^ leading to the development of the theory of vascular calcification in the formation of RPs, which states that the injury and subsequent repair of the vasculature at the renal papillary tip result in atherosclerotic‐like calcification of the vasa recta, eventually causing the formation of plaques that extend to the suburothelium of the papilla.^73^, ^117^ Interestingly, specific physiological properties exist at the tip of renal papilla, including the transition of blood flow from laminar to turbulent and the occurrence of higher osmolality and lower oxygen content, which might contribute to vascular injury and further exacerbate the process.^75^ Similar physiological properties may also be present in Henle loops, leading to cellular injury and calcification reactions, specifically the epithelial‐to‐osteogenic transformation. Several common characteristics have been observed in studies of vascular calcification and renal calcification, including the involvement of reactive oxygen species (ROS) and inflammation, osteogenic transformation, expression of mineralization modulators such as Osteopontin, Matrix Gla protein, fetuin A, osteocalcin and lipids, as well as the involvement of macrophagea and the progression of mineralization.^73^ However, it is important to note that no experimental studies have definitively established vascular calcification as an irreplaceable factor for the calcified plaque formation in renal interstitium.

### 4.2 The growth of calcified plaques in the interstitium

Following CaP nucleation in basement membranes, spherical CaP bodies undergo growth and fusion, eventually forming converged CaP deposits. These deposits continue to grow outward and enter the papilla interstitium, establishing direct contact with collagen fibres and spreading within the collagen framework.^108^ The growth of plaques is facilitated by the mineralization process of collagen.^44^, ^77^ Evan and colleagues discovered that early CaP nucleation occurs in type III collagen of the basement membranes of Henle loops, while the substrate for interstitial growth of the plaque appears to be type I collagen.^25^ Notably, Archana C proposed a novel hypothesis suggesting that mineralization may proceed through non‐classical crystallization mechanisms, initiated by amorphous precursors originating from the polymer‐induced liquid precursor (PILP) process,^87^, ^118^ whereby the presence of negatively charged polymers negatively charged polymers, such as acidic proteins and polysaccharides, in renal tissue leads to the sequestration of calcium ions and phosphate. This sequestration triggers a liquid–liquid phase separation, resulting in the production of CaP amorphous precursors.^87^ Surprisingly, a biomimetic model of Randall's plaque in vitro produces CaP deposits highly similar to those observed in vivo. This model demonstrated that the collagen matrix contributes to promoting and propagating mineralization under conditions similar to the PILP process.^118^

Macrophages play a crucial role in renal tissue inflammation, damage and fibrosis in various acute and chronic kidney diseases.^119^ Since Water and colleagues first discovered that macrophages and multinucleated giant cells are recruited around crystals to clear partial crystals,^120^ macrophages have been implicated in interstitial crystallization in renal papillae.^73^ Taguchi and colleagues^121^ utilized microarrays and immunohistology to demonstrate that genes related to macrophages contribute to the formation of RPs in CaOx stone formers. Considering that CaP deposition in the papilla interstitium involves aberrant mineralization and CaP deposits can be regarded as foreign bodies, interstitial cells surrounding crystals should rapidly response to these foreign bodies by producing chemoattractants, such as MCP‐1 and OPN, to facilitate crystal clearance.^76^, ^122^, ^123^ Kusmartsev and colleagues reported that macrophages treated with recombinant human M‐CSF displayed a stronger capacity for phagocyting crystals than those treated with recombinant human GM‐CSF. Macrophages remove crystals through Clathrin‐dependent pathway.^78^ Another in vitro study demonstrated that M2‐like macrophages (anti‐inflammatory macrophages) exhibited a greater ability to phagocytize CaOx crystals than M1‐like macrophages (proinflammatory macrophages). Analysis of gene expression profiles revealed lower expression of M2‐like macrophages‐related genes in the renal papillae of idiopathic CaOx stone formers compared to controls.^121^ However, chemoattractants induced by crystals may be inadequately secreted, leading to macrophages differentiating into proinflammatory macrophages instead of anti‐inflammatory macrophages or excess recruitment of monocytes, which can exacerbate renal injury within a short period of time, all of which might lead to the failure of crystal clearance. Thus, creating an appropriate immune microenvironment in the renal interstitium to ensure the differentiation of recruited monocytes into M2‐like macrophages is worthy of future investigation.

### 4.3 CaOx stones growth over Randall's plaque

Interstitial plaques in the renal papilla grow slowly and continuously until they extend beneath the urothelium. However, instead of continuing outward growth, the plaques can undergo certain transformations under the influence of physical forces and biochemical factors.^79^ Evan and colleagues discovered that when the plaque comes into contact with urine, it can be covered by organic matrix components present in the urine, such as Tamm–Horsfall protein (THP). This organic matrix might impede the outward growth of CaP deposits and provide a suitable environment for CaOx nucleation.^25^, ^124^ Additionally, CaOx precipitation occurs in an environment with a lower pH than CaP precipitation. As urine undergoes tubular reabsorption and secretion, the renal pelvis urine provides a more suitable pH and supersaturation for CaOx growth. The lower pH of pelvis urine may even cause the dissolution of a small amount of CaP plaque, leading to higher calcium concentration in localized regions.^79^ Furthermore, RPs have a porous structure, and their interfaces are likely not fully closed but permeable. This permeability allows calcium ions to diffuse from the papillary interstitium onto the surface of Randall's plaque, resulting in CaOx precipitation outside the plaques.^125^ Notably, Allison L and colleagues placed biomimetic RPs, as mentioned earlier, into rat's bladders and successfully developed ‘stones’ that closely resembled the stones found in idiopathic CaOx stone formers. They also observed dense accumulation of organic matrix on the surface of the ‘stones’, which intertwined with CaOx aggregates. This finding indicates that organic matrix plays a vital role in the development of CaOx stones.^126^

## 5 THE FORMING PROCESSES OF CALCIFIED AGGREGATES IN RENAL TUBULAR LUMEN

**FIGURE 3:**
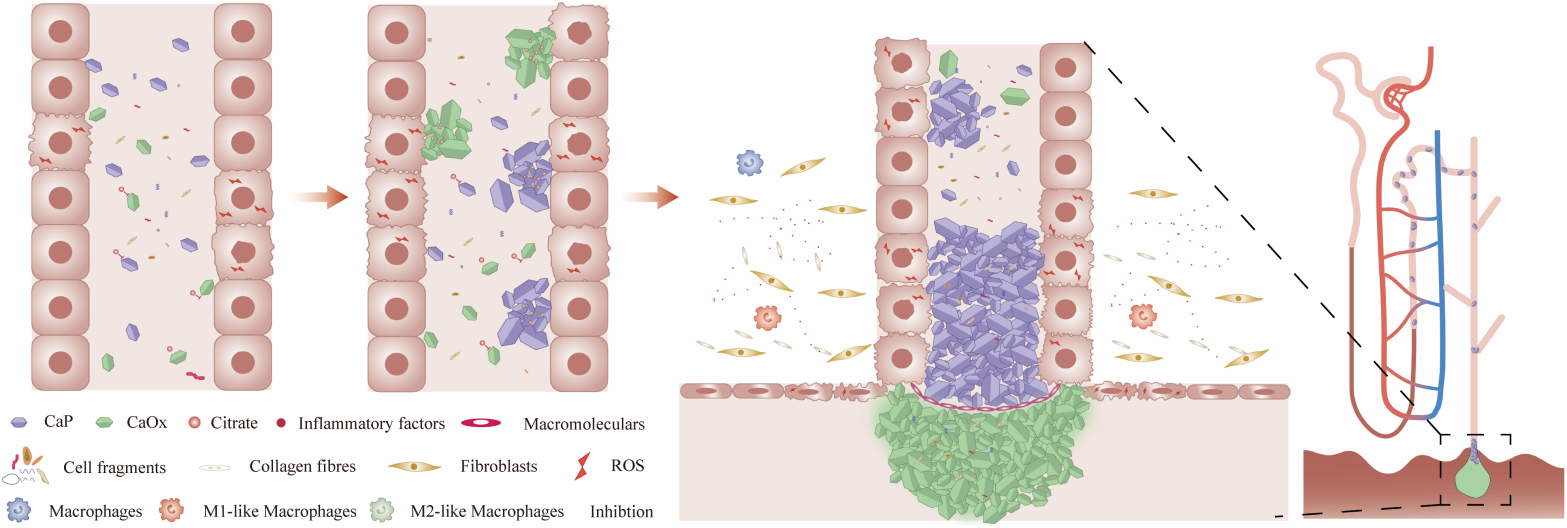
The forming processes of calcified aggregates in renal tubular lumen. Under the conditions of abnormal urine, the forming processes of calcified aggregates in renal tubular lumen mainly undergoes crystal nucleation, growth, aggregation and adhesion. Cell‐crystal interaction, such as conditions like the presence of CaOx and CaP crystals, can lead to RTECs producing excessive ROS, resulting in cellular damage. This damage can trigger a cascade of events, leading to the production of proinflammatory cytokines and the initiation of inflammatory responses in the interstitial tissue. In addition, this damage results in the release of cell fragments into tubular lumens, which then bind to CaP and CaOx crystals. Finally, the calcified aggregate eventually grows into large aggregates to block the opening of the collecting duct and is exposed to the urine of calcium oxalate supersaturation, leading to the formation of CaOx stones, wherein macromolecules play a role in modulating crystal aggregation, growth and adhesion. In addition, citrate has been shown to inhibit the nucleation and growth of crystals.

The calcified aggregates, blocking the openings of the Bellini ducts (also termed Randall's plug), are commonly observed in patients with various types of kidney stones, including CaP, uric acid stones, struvite and cystine stones, as well as in conditions such as primary hyperoxaluria, hyperparathyroidism and bariatric surgeries. These calcified aggregates are also frequently found in animal models of hyperoxaluria‐induced kidney stones.^121^ Notably, even in cases of idiopathic CaOx stones, a small portion of stones may be induced on the calcified aggregates, which are composed of intratubular CaP deposits.^90^ Multiple lines of evidence from optical and electron microscopy examinations have shown a close association between the presence of Randall's plugs and intratubular deposits of extensive crystals, focal injury and death of renal tubular epithelial cells, as well as renal interstitial inflammation and fibrosis.^53^, ^81^ In the context of urinary supersaturation with stone‐related solutes, crystals undergo nucleation, growth, aggregation and adhesion, aided by various modulators. Eventually, these crystals form large aggregates. When these aggregates block the openings of the Bellini ducts, they form Randall's plugs. Over time, these calcified aggregates are continuously exposed to aberrant urine, leading to stone growth over the Randall's plugs, similar to the growth observed over RPs described earlier (Figures 1B and 3).^25^, ^79^

### 5.1 Crystal nucleation

Nucleation is the process by which solute molecules in a solution come together to form visible microscopic crystals with a characteristic lattice structure that is insoluble. In the context of stone formation, urine supersaturation is the driving force for the nucleation of stone‐related salts.^40^ For instance, when the supersaturation of calcium oxalate in urine reaches 7–11 times its solubility, nuclei of soluble calcium oxalate begin to form.^37^ Heterogeneous nucleation is more likely to occur as the mechanism for nuclei forming, as it requires lower solute concentrations than homogeneous nucleation.^7^, ^38^ Heterogeneous nucleation involves the assistance of other substances, such as urinary organic matrix, proteins or crystals of other solutes, membrane‐bound vesicles, lipids and cell‐degrading products. These substances act as nucleators, facilitating the formation of nuclei.^38^, ^40^ Therefore, the notion that injured epithelial cells serve as the origin of stone formation has been recognized by many scholars.

CaOx can exist in several forms, including CaOx monohydrate (COM), CaOx dihydrate (COD), CaOx rihydrate (COT) or amorphous CaOx (ACO), wherein COM is the most common and annoying form.^127^, ^128^, ^129^ Crystal nucleation may occur through classical and non‐classical pathways.^68^ Classical crystal nucleation involves the formation of a crystalline core through the adherence of monomer units, leading to the formation of rhombohedral COM, which has the highest thermodynamic stability. On the other hand, rectangular COM crystals nucleate through non‐classical pathways, which may involve the formation of oligomeric or polymeric nanocrystals and amorphous precursors. Notably, Ruiz‐Agudo and colleagues reported that citrate could interfere with the nucleation of CaOx by slowing down the formation of amorphous calcium oxalate (ACO) and its conversion into the crystal phase.^68^ Citrate could also increase the number of water molecules attracted to calcium ions when they enter the crystal lattice, leading to instability in the CaOx structure.^128^ Another in vitro study utilizing atomic force microscopy (AFM) and high‐resolution transmission electron microscopy (HRTEM) found that different forms of CaOx crystals can be developed in solutions with varying Ca/Ox molar ratios. In solutions with a low Ca/Ox molar ratio, pre‐nucleation nanoparticles of approximately 1.03 nm in size form and eventually develop into hexagonal COM crystals. Conversely, solutions with a high Ca/Ox molar ratio result in pre‐nucleation nanoparticles of approximately 1.99 nm, eventually evolving into quadrangular‐pyramid‐shaped COD crystals. Additionally, COD crystals can spontaneously dehydrate and convert into COM crystals in solutions with a high Ca/Ox molar ratio.^129^ Therefore, controlling the pathways of crystal nucleation can potentially regulate the characteristics of CaOx crystals, including their crystal structure, morphology and hydration degree, which may aid in preventing the formation of CaOx stones.

### 5.2 Crystal Growth and Aggregation

Once crystal nuclei form in the renal tubules, the growth of crystals occurs through the addition of new crystal components in urine and the aggregation of already formed crystals. Both processes occur simultaneously and complement each other to form larger and harder stones.^7^, ^38^, ^40^ Crystal growth can occur not only on the tubular walls where the nuclei are attached but also directly in free solution.^7^ Endoscopic examination found that round and unattached microliths appeared at dilated inner medullary collecting ducts (IMCD) in patients with cystine stones,^130^ while the feasibility of crystal growth in a free solution is a topic of debate and requires higher supersaturation and a more suitable pH environment. Urine supersaturation of stone‐related salts is the primary driving force for crystal growth, but it is not the sole factor.^25^ The organic matrix, which makes up approximately 2%–3% of the dry weight of the stone, plays a significant role. The role of macromolecules in the kidney stone formation has been extensively reviewed by researchers.^38^, ^40^, ^73^ For example, urine macromolecules act as a decisive player during crystal growth and aggregation,^131^, ^132^ which could be promoted by matrix substance A, and other uncharacterized urinary proteins. Some macromolecules have been reported to slow or halt crystal growth and aggregation, such as glycosaminoglycans, nephrocalcin, inter α inhibitor and calgranulin, wherein Tamm–Horsfall protein and osteopontin may have dual roles.^7^ In 2016, Chung and colleagues^69^ published an remarkable article in *Nature* demonstrating that hydroxycitrate, like citrate, can inhibit the nucleation and growth of calcium oxalate monohydrate. The researchers proposed that for a substance to have the potential ability to dissolve crystals, it must meet the criterion: BE_inhibitor‐crystal_ >> BE_solute‐crystal_ (BE represents Binding Energy). More importantly, the ability of a substance to inhibit stone growth can be predicted using density functional theory (DFT), by analysing the adsorption energy on the COM (100) surface, the binding energy on COM (021) surface and the strength of binding with the COM (100) and (021) surface.^69^ A more general insight is that these inhibitors of COM growth all possess a large number of anionic groups, and their inhibitory activity increase as the number of anionic groups increases. Additionally, the combined effects of different macromolecules on growth inhibition in urine require further investigation.^39^ Similar to the treatment of cystine stones with L‐CDME and α‐Lipoic acid,^133^ Shtukenberg and colleagues proposed the concept of designing tailor‐made additives that can precisely recognize the specific crystal structure, promote step pinning and block step advancement. This approach can efficiently hinder crystal growth and prevent stone formation.^134^

Scientific calculations suggest that the process of crystal grow alone is unlikely to result in formation of pathologically relevant‐sized stones. Crystal aggregation, facilitated by Van der Waal's forces (VWF) among particles, is considered essential in stone formation.^40^ CaOx crystals typically have a negatively charged surface, which results in electrostatic repulsion between individual crystals and prevents their aggregation. However, when urinary supersaturation of calcium oxalate occurs, it can neutralize the negative charge on the crystal surface, leading to aggregation without electrostatic repulsion. The presence of macromolecules adhering to the crystal surface can also contribute to the lack of electrostatic repulsion and enhance aggregation.^131^ Previous studies have demonstrated that polyanions can decrease the aggregation of COM crystals. In contrast, polycations have weaker inhibitory capabilities and may even promote crystal aggregation.^132^

### 5.3 Crystal adhesion and retention

The attachment of crystals to the walls of renal tubules or their blockage in tubular lumens is known as crystal adhesion or retention. Given that the tubular fluid only takes a short time (5–7 min) to pass through the nephron, this timeframe is insufficient for crystals to grow and aggregate into obstructions that block renal tubules.^75^ Therefore, crystal adhesion is considered a crucial step in stone formation, exerting its synergistic effect at any crystallization stage. In the collecting ducts, the combination of peristaltic waves and the physical structure, with the opening of the ducts narrower than the duct lumen, leads to turbulent urine flow, which may contribute to crystal retention and the formation of Randall's plugs.^7^, ^79^ Currently, a significant portion of research on the aetiology of stone formation focuses on crystal adhesion, also known as crystal‐cell interaction. Numerous studies have demonstrated that the primary prerequisite for crystal adhesion is the presence of abnormal urine, such as conditions characterized by hypercalcinuria, hyperoxaluria, hyperuricuria and crystalluria, which can cause injury to epithelial cells.^38^, ^135^, ^136^, ^137^

Injured cells can express specific molecules on their surface, such as hyaluronic acid, phosphatidylserine and CD44,^138^, ^139^ and promote membrane flip to expose negatively charged components to urine. These components, including annexin A1, annexin A2, α‐enolase, HSP70 and HSP90,^140^, ^141^ both of which serve as sites of crystal adhesion.^136^, ^142^ In addition, the exposed basement membrane and the surface of replenished new cells favour crystal adhesion.^77^ Vinaiphat and colleagues demonstrated that PMCA2 acts as a receptor for COM crystals and is involved in crystal adhesion.^143^ Zhang and colleagues reported that succinate can inhibit crystal adhesion, thus preventing stone formation.^144^ Mulay and colleagues discovered that TNFR signalling actively contributed to the initiation of CaOx crystal adhesion, and blocking TNFR therapeutically may slow down the process of stone formation.^145^ Urinary macromolecules, such as fibronectin,^146^ osteopontin and Tamm–Horsfall protein,^147^ have dual roles in modulating crystal adhesion. Some studies have identified potential treatment strategies to inhibit crystal adhesion, including using various antioxidants, caffeine,^141^ Trigonelline,^142^ Costus arabicus^148^ and Porphyra yezoensis Polysaccharide.^96^, ^97^ Additionally, a study on traditional Chinese medicines proposed that efficient inhibitor molecules should have hydroxyl or amine groups linked with C2O4^2−^ and carboxyl and phenolic hydroxyl groups connected with Ca^2+^ on the (100) and (010) surfaces of COM crystals. It was found that inhibitors meeting certain characteristics, such as pKa < 3.5, logD (pH = 6) < 0 and H‐number > 0.1 mmol, possess stronger activity in inhibiting crystal growth and adhesion.^149^

## 6 EXPERIMENTAL MODELS

The complexity of stones formation, with its diverse components, multiple pathogenic factors and intricate mechanisms, poses significant challenges for research in this field. Recognizing the disease and establishing experimental models form a mutually beneficial cycle, as establishing appropriate models allows for the dissection of complex mechanisms into manageable modules, facilitating scientific investigation. Therefore, selecting and developing suitable experimental models are crucial in this regard. In many published studies, human renal tubular epithelial (HK‐2) cells, Madin‐Darby Canine Kidney (MDCK) cells and normal rat kidney epithelial‐like (NRK‐52E) cells have been used for cell experiments. These cells are often co‐incubated with CaOx crystals, high concentrations of oxalate or sodium oxalate to perform the cell experiments.^150^, ^151^, ^152^ Macrophage research has primarily focused on macrophage polarization and their phagocytic response to CaOx crystals.^78^, ^153^ However, it is important to note that CaP is not only a major component of interstitial plaques, but also a prominent participant in the intratubular portion of Randall's plugs, suggesting that the response of RTECs to CaP crystals may occur earlier than their response to CaOx crystals.^79^ Therefore, it is crucial to explore the interactions of various cells with CaP crystals. Furthermore, since RTECs from different parts of the nephron have distinct functions, they may exhibit different responses to abnormal urine.^38^ Therefore, investigating different mechanisms in specific cell types from different nephron regions is essential for gaining a comprehensive understanding of stone formation.

To data, ethylene glycol (EG)‐induced rat models have been widely utilized as animal models to investigate the pathogenesis of nephrolithiasis. These models can be easily and rapidly induced to develop hyperoxaluria and deposit calcium oxalate crystals in tubular lumen.^75^ However, it should be noted that these rat models may not fully replicate the pathogenesis of stone formation in humans. For instance, the deposition of calcium oxalate crystals in renal tubular lumens is observed in only a minority of stone formers, such as patients with PH1. Moreover, stone formation is typically a chronic pathological process, whereas these models represent relatively acute crystal damage. Most importantly, these models do not fully simulate the most common pathological process, which is the formation of RPs.^25^ With the advancement in the study of genetic factors related to kidney stone formation, several mouse models with specific gene knockout have been successfully developed. These mouse models can induce intratubular and interstitial crystal deposition simultaneously, including NPT2^−/−^, UMOD^−/−^, OPN^−/−^, NHERF1^−/−^, ABCC6^−/−^ and CLDN2^−/−^ knockout mouse.^25^, ^73^, ^102^ These gene knockout mouse models hold great potential in advancing our understanding of the mechanisms underlying stone formation and taking the research to a new level. Therefore, it is essential to accelerate the identification of candidate genes and loci in humans, which can then be studied using mouse models to establish appropriate models for investigating stone formation.

In addition, the nephrolithiasis model of Drosophila melanogaster has been used by Chen and colleagues. This model utilizes the Malpighian tubules of Drosophila, which have similar functions to human renal tubules in terms of absorption and secretion. The accessibility of these tubules makes them suitable for studying nephrolithiasis.^154^ Furthermore, Archana and colleagues employed decellularized porcine kidneys to construct a biomimetic Randall's plaque, which was then implanted into rats' bladders to investigate the mechanisms of stone formation on RPs. This approach provides a better simulation of the stone formation process.^118^, ^126^ It is worth noting that while current research often relies on artificially induced stone formation models, there is potential for gaining valuable insights from naturally occurring stone formation in large‐animal models. Animals such as dogs, cats, otters and pigs, which naturally develop stones, could provide important information for enhancing our understanding of stone pathogenesis and should be given more attention in future studies.^155^

## 7 CONCLUSIONS AND FUTURE PERSPECTIVES

After nearly three decades of development in this field, significant progress has been made, revealing a general framework of the stone formation process. However, the exact underlying mechanism remains elusive. Many scholars consider stone formation as a metabolic disease akin to hypertension and diabetes, where the stone itself is the end product resulting from long‐term pathological reactions,^31^, ^115^ As such, lithotripsy may provide temporary relief rather than a permanent cure. In addition, we have a guess: the stones that surgeons are dealing with may be just the tip of the iceberg. The products of formation processes of calcareous nephrolithiasis in renal interstitium and tubule lumen are the basic for the recurrent appearance of the detectable kidney stones. If the lesions within renal interstitium and tubule lumen still exist, the stones will continue to grow relying on these lesions with the aid of abnormal urine and various modulators.

Given its chronic nature, future research should focus on extensive epidemiological studies and randomized controlled trials to identify reliable risk factors for stone formation and specific biochemical markers present in blood and urine, which not only aids in diagnosis, but also enhances our understanding of nephrolithiasis. Furthermore, there may be commonalities between metabolic diseases, such as the involvement of oxidative stress and inflammatory responses, which warrant further investigation.^92^ Although numerous studies have explored intracellular molecular processes induced by cell‐crystal interactions, the development of molecular‐targeted drugs has been elusive. The intricate signalling pathways triggered by these interactions present complex challenges that require the identification of a common trigger. Therefore, the unique phenomenon of kidney stones, including crystallization and mineralization, cannot be disregarded.

Interdisciplinary research involving urologists, pathophysiologists, chemists and other basic scientists is crucial to prevent stone formation by targeting early processes such as crystal nucleation, deposition, growth, aggregation and cell‐crystal interactions. It is important to note that a comprehensive understanding of kidney pathology in patients with nephrolithiasis is essential to advance this field beyond the findings from experimental models alone. Therefore, when ethical standards are met, closer examination of human renal tissue samples, particularly at the earliest stages of plaques, can provide valuable insights into stone formation. Overall, various mechanisms have been proposed to explain observations in both human and experimental models, each contributing significantly to the aetiology of urolithiasis. However, uncovering the mystery of mineral formation within the renal milieu and developing a unified and definitive theory of stone formation pathogenesis remains the ultimate achievement in this field.

## AUTHOR CONTRIBUTIONS

**Caitao Dong:** Data curation (equal); writing – original draft (lead). **Jiawei Zhou:** Data curation (supporting); visualization (equal). **Xiaozhe Su:** Data curation (equal); writing – original draft (supporting). **Ziqi He:** Data curation (supporting); investigation (lead). **Qianlin Song:** Conceptualization (lead); formal analysis (lead). **Chao Song:** Conceptualization (supporting); formal analysis (supporting). **Hu Ke:** Investigation (supporting); visualization (equal). **Chuan Wang:** Visualization (equal). **Wenbiao Liao:** Resources (equal); writing – review and editing (supporting). **Sixing Yang:** Resources (equal); supervision (lead); writing – review and editing (lead).

## FUNDING INFORMATION

The study was funded by three projects, including Natural Science Foundation of China (82270797), Natural Science Foundation of China (82070723) and Natural Science Foundation of Hubei Province, China (2022CFC020).

## CONFLICT OF INTEREST STATEMENT

All authors declare no conflicts of interest in this study.

## Data Availability

Not applicable.
